# Regulation of Mammalian Physiology by Interconnected Circadian and Feeding Rhythms

**DOI:** 10.3389/fendo.2017.00042

**Published:** 2017-03-08

**Authors:** Florian Atger, Daniel Mauvoisin, Benjamin Weger, Cédric Gobet, Frédéric Gachon

**Affiliations:** ^1^Department of Diabetes and Circadian Rhythms, Nestlé Institute of Health Sciences, Lausanne, Switzerland; ^2^Department of Pharmacology and Toxicology, University of Lausanne, Lausanne, Switzerland; ^3^School of Life Sciences, Institute of Bioengineering, Ecole Polytechnique Fédérale de Lausanne, Lausanne, Switzerland; ^4^School of Life Sciences, Ecole Polytechnique Fédérale de Lausanne, Lausanne, Switzerland

**Keywords:** circadian rhythm, liver, metabolism, feeding behavior, genomics, proteomics

## Abstract

Circadian clocks are endogenous timekeeping systems that adapt in an anticipatory fashion the physiology and behavior of most living organisms. In mammals, the master pacemaker resides in the suprachiasmatic nucleus and entrains peripheral clocks using a wide range of signals that differentially schedule physiology and gene expression in a tissue-specific manner. The peripheral clocks, such as those found in the liver, are particularly sensitive to rhythmic external cues like feeding behavior, which modulate the phase and amplitude of rhythmic gene expression. Consequently, the liver clock temporally tunes the expression of many genes involved in metabolism and physiology. However, the circadian modulation of cellular functions also relies on multiple layers of posttranscriptional and posttranslational regulation. Strikingly, these additional regulatory events may happen independently of any transcriptional oscillations, showing that complex regulatory networks ultimately drive circadian output functions. These rhythmic events also integrate feeding-related cues and adapt various metabolic processes to food availability schedules. The importance of such temporal regulation of metabolism is illustrated by metabolic dysfunctions and diseases resulting from circadian clock disruption or inappropriate feeding patterns. Therefore, the study of circadian clocks and rhythmic feeding behavior should be of interest to further advance our understanding of the prevention and therapy of metabolic diseases.

Most living organisms are subjected to daily environmental changes imposed by the 24-h rotation of the Earth around its own axis. To anticipate these environmental variations, organisms developed a self-sustained timekeeping system, called the circadian clock (from the Latin *circa* and *diem* meaning “about a day”), which regulates behavior and physiology. The mammalian clock is organized in a hierarchical manner by a master pacemaker located in the suprachiasmatic nucleus (SCN) of the hypothalamus. This synchronizes subsidiary peripheral oscillators present in nearly every cell of the body (1). At the molecular level, circadian rhythms in gene expression are generated by interconnected transcriptional and translational feedback loops (TTFLs), in which multiple layers of control, including temporal transcriptional, posttranscriptional, and posttranslational regulation, play important roles (2, 3).

The discovery of the prominent role of the SCN for circadian rhythmicity originates from extensive lesion studies reporting a region in the anterior hypothalamus necessary for rhythmic locomotor activities (4). Moreover, circadian rhythms were partially restored by transplantation of fetal SCN tissue in SCN-lesioned animals and also in genetically engineered clock-deficient animal models. In addition, SCN-lesioned hosts displayed altered locomotor activities following SCN transplantation from arrhythmic mutant mice (5, 6). The SCN regulates the daily adaptation of the internal clock to environmental light–dark cycles. Mammals perceive light information through the retina, where photoreceptors [termed intrinsically photosensitive retinal ganglion cells (ipRGCs)] express melanopsin, a photopigment that transmits the information directly to the SCN through the retinohypothalamic tract (7, 8). In addition, ipRGCs receive non-visual cues from rod and cone photoreceptors and transmit these to the SCN, showing their central role in photic input processing (9, 10).

As a master clock, the SCN provides robustness and plasticity to the circadian system. Indeed, the SCN exhibits an adaptive response to photoperiod lengths, as shown by the opposite consequences of exposures to varying photoperiods upon SCN oscillations (11). This flexibility of daily resetting may reside in the intracellular coupling of greatly heterogeneous SCN neurons (12, 13). Indeed, the mammalian SCN is composed of ~20,000 neurons, which contain a circadian clock (14) but exhibit a broad range of phases and periods of neural firing when isolated *in vivo* or in cell culture experiments (15). Furthermore, SCN neuron explants and high density cultures of SCN neurons produced robust synchronized neuronal firing, even when the circadian clock had been genetically altered (12, 13). At the cellular level, photic cues entrain the circadian clock through several signaling pathways. Notably, light pulses triggered during the dark phase induce the expression of the circadian clock *Period* (*Per*) genes through the activation of the extracellular signal-regulated kinase (ERK) pathway (16, 17). Interestingly, this occurs exclusively when light is provided during the dark phase, suggesting that *Per* genes are involved in night–day transition.

## The Molecular Circadian Clock

As mentioned, the molecular clock has been conserved throughout evolution and works through TTFL (18). The circadian clock is also targeted by multiple posttranslational modifications that increase the robustness of the oscillatory system by fine-tuning the localization and degradation of core oscillator proteins (2). Notably, proteasome-mediated degradation of core circadian proteins is necessary for the rhythmic expression of core clock genes (19).

**Figure 1:**
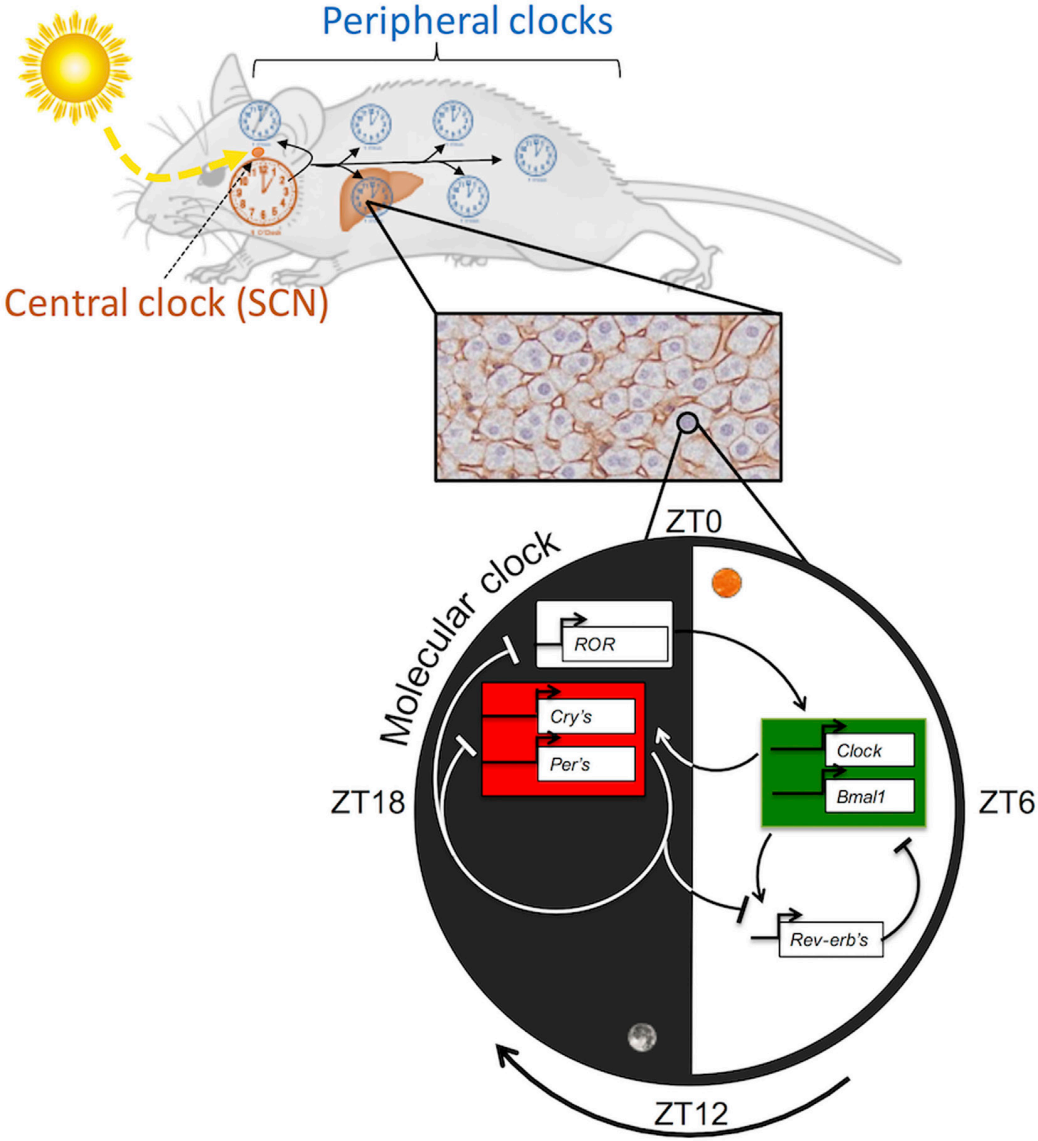
**Hierarchical organization of the circadian clock in mammals**.

In mammals, the Circadian Locomotor Output Cycles Kaput (CLOCK) and Brain and Muscle ARNT Like protein 1 (BMAL1) proteins, two transcription factors belonging to the family of bHLH-PAS (basic helix-loop-helix; Per-Arnt-Sim domain) proteins, enhance the positive limb of the TTFL. CLOCK and BMAL1 heterodimerize and initiate transcription by binding to specific DNA elements like E-box-related motifs (5′-CACGT[G/T]) in the promoters of target genes, including the *Per* and *Cryptochrome* (*Cry*) paralogs. Subsequently, PER and CRY accumulate and dimerize in the cytoplasm and then translocate into the nucleus to inhibit the transcriptional activity of the CLOCK:BMAL1 heterodimer, resulting in the downregulation of their own expression (3). Another primordial loop operates to stabilize the molecular core oscillator by connecting it to metabolic effectors (20). This loop is composed of other targets of the CLOCK:BMAL1 heterodimer such as the nuclear receptors retinoic acid-related orphan receptor α (RORα) and reverse erythroblastosis virus α (REV-ERBα or NR1D1), which respectively activate and repress *Bmal1* expression by binding response elements (RORE) present in the *Bmal1* promoter (21, 22) (Figure 1). An additional feedback loop involving the bHLH proteins DEC1 (or BHLHE40) and DEC2 (or BHLHE41) plays a role in rhythmic metabolism by regulating BMAL1 activity through competitive binding to its cognate sites (23, 24). In addition, the circadian clock controls the rhythmic expression of the PARbZip transcription factors DBP, HLF, and TEF and their repressive counterpart E4BP4 (or NFIL3). These factors are not directly involved in clock regulation but play an important role in the regulation of metabolism and physiology by the circadian clock (25).

## Synchronization of Peripheral Clocks by the SCN and Its Downstream Feeding Rhythms

Principally, SCN output signals are mediated by circadian variation of neuronal firing and transmitter release at SCN axons (13). Neuronal connection appeared as a major effector of the SCN control since surgical isolation of the SCN (which did not compromise SCN rhythms) resulted in the abolition of circadian rhythms in other brain regions (26). Peripheral organs also contain endogenous sustain oscillators as shown by *ex vivo* cultures of liver, lung, and skeletal muscle tissues (27). These oscillations in peripheral organs progressively dampened and were desynchronized after SCN lesion, suggesting that the SCN coordinates peripheral clocks to “tick” properly (28). However, local clocks are also necessary for circadian functions. Notably, the specific inactivation of the local oscillator in adipocytes, pancreatic islets, and the liver resulted in an alteration of lipid, insulin, and glucose homeostasis, respectively (29–31). Conversely, mice with a conditionally active liver clock lost daily variation of most liver transcripts following REV-ERBα overexpression and suppression of clock oscillation (32). However, several transcripts like *Per2* still exhibited robust oscillations, suggesting that besides a functional hepatocyte clock, systemic cues can drive hepatic rhythms independently. Indeed, the SCN fashions peripheral clock rhythmicity through the modulation of systemic cues such as hormones, body temperature, and feeding behavior (33–36).

Interestingly, feeding behavior interacts with both temperature and humoral synchronization of peripheral clocks. In most mammals, feeding behavior exhibits a pronounced circadian rhythmicity characterized by major food consumption during the active phase. Nocturnal rodents consume 70%–80% of their total daily food intake during the active dark phase. Lesion of the SCN led rats to eat similar proportions of food during the light phase and the dark phase, showing that a functional master clock is necessary for proper feeding behavior (37). Conversely, genetic alteration of the molecular clock impaired the rhythmic food consumption in various whole-body circadian mutant models (31, 38, 39). Daily feeding–fasting cycles were shown to be primordial *Zeitgebers* (for time givers) for peripheral oscillators by shifting mealtimes to the resting period. This inverted feeding regimen rapidly inverted peripheral clocks in wild-type (WT) mouse liver, kidney, heart, and pancreas but had little to no effect on the central oscillator (36, 40). The food induction of phase shifting occurs in both light–dark and constant darkness conditions, indicating that entrainment of peripheral clocks under restricted feeding conditions may occur independently of the SCN. Indeed, the arrhythmic expression of *Per1* and *Per2* genes in the liver of SCN-lesioned mice was restored when food was restricted to a 4-h time window during the light phase (41). In addition, food restriction can partially restore rhythmicity of hepatic gene expression in mouse models with a defective circadian clock (39). However, food-induced shifting of peripheral clocks occurs progressively, and 12-h inversions of liver oscillations need slightly more than 1 week to be effective (36). Recently, the investigation of *in vivo Bmal1*-luciferase expression showed similar results (42). In addition, the authors revealed that entrainment of the liver clock by inverted feeding occurred rapidly in mice with ablated SCN. Therefore, the SCN may counteract peripheral clock uncoupling imposed by inverted food regimens, potentially through the rhythmic secretion of glucocorticoids (43).

Interestingly, the adrenal gland is connected by a polysynaptic pathway to the SCN, which controls the daily release of glucocorticoids (44). The SCN stimulates the daily release of corticosterone in a light-dependent manner, leading to a glucocorticoid surge during the light phase, which reaches maximum levels at the day–night transition, anticipating the active/feeding phase in nocturnal rodents (45). Glucocorticoids may be a way for the SCN to specify rhythms of peripheral clocks and to delay a phase shift under inverted feeding conditions. Indeed, adrenalectomized animals harbored fast food-induced resetting of peripheral clocks, similarly to SCN-ablated mice (43). Glucocorticoids act on peripheral clocks through the interaction with glucocorticoid receptors, which bind glucocorticoid response elements in the promoters of target genes. These regulatory elements have been found in the promoter of core clock genes such as *Bmal1, Cry1, Per1*, and *Per2* (46–48), showing the strong interconnection between the two systems (49). Conversely, glucocorticoids were shown to entrain peripheral clocks, as suggested by the ability of dexamethasone (a glucocorticoid analog) to trigger oscillations in rat fibroblasts (33).

In parallel, temperature modulation was proven to sustain and synchronize peripheral oscillators (34, 50, 51). Indeed, temperature fluctuations impact circadian periods in fibroblasts as shown in fibroblasts expressing *Bmal1*-luciferase reporters. Short periods were associated with higher temperatures, whereas an opposite effect was observed with lower temperatures (52). Interestingly, temperature compensation was dampened in *Per1* KO fibroblasts, which exhibited similar oscillations to control counterparts (52). In addition, the heat shock factor 1 is likely involved in temperature-mediated modulation of the liver clock (53). Conversely, in SCN-lesioned animals, food restriction induced both rhythmic locomotor activities and temperature rhythms (54, 55). Finally, temperature could also impact rhythmic gene expression through the regulation of mRNA splicing efficiency, as recently demonstrated for cold-inducible RNA-binding protein (56).

Restriction of feeding to a few hours during the resting phase enhances oscillation of metabolic factors including glucose, free fatty acids, and glucocorticoids (57). Similarly, this short supply of food during the day quickly alters daily behavioral rhythms such as locomotor activity to anticipate food availability (55, 58). This food-anticipatory activity (FAA) persists when mice are subsequently placed under food deprivation. Interestingly, most of the murine models with a defective circadian clock presented normal FAA (58–60). Entrainment to food can also occur in rodents with SCN lesions, indicating that the neuronal locations governing FAA are at least partially distinct from those who participate in light entrainment (54, 58).

Although the brain regions involved in FAA still need to be discovered, several studies suggest that peripheral organs participate in FAA through humoral routes. Notably, Ghrelin-secreting cells of the stomach were shown to constitute potential food-entrainable oscillators. Ghrelin stimulates food intake during feeding restriction, and Ghrelin receptor knockout animals show a reduction in FAA (61, 62). In addition, the gut-secreted oxyntomodulin is also involved in the synchronization of the circadian clock through feeding cues (63). In parallel, *Per2* has recently been shown to mediate hepatic action upon FAA. Although *Per2* mutation in the whole body is known to impair food anticipation in mice, the liver-specific *Per2* mutation (L-*Per2*) is sufficient to disrupt this circadian behavior (64, 65). Under inverted feeding conditions, PER2 modulates *Cpt1a* and *Hmgcs2* expression, two rate-limiting enzymes for β-hydroxybutyrate synthesis. Interestingly, β-hydroxybutyrate injection rescues FAA in L-*Per2* mice, which provides a way for the liver to participate in adaptation of feeding behavior. Another example is given by the adipocyte clock, which is involved in daily leptin secretion (30). Leptin reduces appetite, and its signaling is blunted in circadian clock-mutant animals and under chronic jetlag (66). Adipocyte-specific deletion of *Bmal1* resulted in the impairment of leptin levels in plasma, as well as defective feeding behavior. The regulation of feeding behavior appears to integrate multiple layers of control involving not only the central clock but also clock-independent food-entrainable oscillators employing central and peripheral organs.

## Transcriptional Control of Circadian Output Genes

Recently, the transcriptional landscape of circadian core clock transcriptional regulators has been revealed by time-resolved Chip-seq experiments (67). The DNA-binding preference for circadian activators (BMAL1, CLOCK, and NPAS2) and repressors (PER1, PER2, and CRY2) showed opposite phase specificity. DNA binding of circadian transcriptional regulators showed accompanying rhythms of histone modifications, indicating that rhythmic fluctuations of liver transcripts partially emerge as a result of transcriptional regulation (67, 68). Consequently, an important part of the liver transcriptome exhibits daily oscillations connected to genes encoding proteins involved in metabolic regulations (69–71). Conversely, several aspects of glucose and lipid metabolism are altered in circadian-deficient mice models (1).

As mentioned before, additional feedback loops connect the core loop to metabolic regulations. One connection is explained by the interaction of PER2 with nuclear receptors REV-ERBα, PPARα, and PPARγ involved in both glucose and lipid metabolism regulation (72, 73). These interactions likely specify oscillations of genes targeted by nuclear receptors. REV-ERBs have a dual role in stabilizing the core loop by binding RORE in their promoter and driving metabolic gene expression through their interaction with other transcription factors (74, 75). A secondary connection is the interaction of CRY proteins with glucocorticoid receptors, linking CRY to glucose metabolism (76). On the other hand, PPARα is activated by binding to fatty acids and regulates glucose and lipid metabolism. Importantly, PPARα activity is also indirectly controlled by the circadian effectors DBP, TEF, and HLF. Indeed, mice lacking these three PARbZip family members showed impaired hepatic fatty acid content, due to the loss of oscillations of rate-limiting enzymes involved in FA synthesis (77). Interestingly, PPARα activity could be rescued in PARbZip KO mice through the stimulation of *de novo* fatty acid synthesis induced by a fat-free diet. Another transcription factor, SREBP1, is controlled by the circadian clock and food inputs. Indeed, SREBP1-mediated transcription is altered in *Bmal1* and *Rev-erb*α KO mice (78, 79). Conversely, day time food-induced resetting of the clock in WT mice led to a 12-h phase shift of SREBP1 activation (80) and rescued its rhythmic activity in *Cry1/Cry2* KO mice (39).

Contradicting these numerous examples of circadian clock-mediated transcriptional regulation, some studies suggested that a minor proportion of rhythmic transcripts were driven by transcriptional events (67, 81). In contrast, investigation of DNA-binding dynamics of the DNA polymerase II revealed a higher importance of transcriptional regulation in guiding mRNA oscillations (82). Similarly, we observed that most of the cyclic mRNA accumulation originated from rhythmic transcriptional events (83). These discrepancies may originate from differences in the analysis (84) or nature of the data. Indeed, experimental conditions such as light–dark schedule or constant darkness, as well as *ad libitum* or night-restricted feeding, play an important role in the quantitative rhythmic transcriptome. This is suggested by the consolidation of mRNA rhythms in mice subjected to night or day feeding restrictions (39, 85). Still these studies univocally demonstrate that posttranscriptional regulations are at least partially involved in circadian rhythmicity.

Indeed, transcriptional regulations are not necessarily reflected at the proteomic level. Early proteomic-based investigations showed that rhythmic variation of protein abundance is not fully explained by variation in mRNA levels (86). More recent studies with higher coverage of mouse liver and SCN also concluded that about half of the rhythmic proteins are encoded by non-rhythmic mRNA (87–89). Considering rhythmic protein contents in specific liver organelles such as nuclei and mitochondria, correlation between protein and mRNA levels is even worse, which suggests that rhythmicity likely results from cell trafficking (90, 91). Although protein secretion has been suggested as a possible explanation for rhythmic liver protein contents (89), other processes like mRNA translation could be also involved.

## The Impact of Circadian and Feeding Rhythms on mRNA Translation

The first evidence of a major role of mRNA translation in the generation of circadian rhythms came from the study of the rhythmic photosynthesis of the giant green algae *Acetabularia*. *Acetabularia* has a single nucleus located in the rhizoid, which allows the regeneration of the cell if its cap is completely removed. However, not only can the cell survive for several weeks without its nucleus, but the rhythmic photosynthesis of the plant continues under this condition (92). In addition, studies examining the reintroduction of an out-of-phase nucleus into the plant showed that the clock in the cytoplasm determines the phase of the rhythmic photosynthesis. The cytoplasmic clock entrains the nuclear clock, which suggests that the latter has a minimal effect on this rhythm (93, 94). Further experiments show that the rhythmic synthesis of a subset of proteins is dependent on the translation machinery, demonstrating for the first time the circadian translation of mRNA (95).

Secondary evidence came from the study of the luminescent unicellular dinoflagellate *Gonyaulax*, which presents circadian photosynthesis, motility, cell division, and luminescence (96). The nocturnal luminescence of *Gonyaulax* is produced by a complex of three proteins, whose synthesis is controlled by the circadian clock at a posttranscriptional level (97, 98). Further experiments show that this translational regulation is controlled by the UG-repeat sequence binding protein CCTR, which rhythmically binds the 3′-untranslated region (UTR) in the RNA of this luminescent protein and represses its expression during the day (99). In addition to these historical discoveries in unicellular organisms, recent evidence suggests that circadian clock-regulated translation also occurs in mammals.

The first observation suggesting a rhythmic translation in mammals is the description of a rhythmic polysome profile in rat liver, with around 25% more polysomes present during the dark phase than during the light phase (100). This observation was later confirmed by electron microscopy experiments, which showed that the polysomal volume density is four times higher at dusk than at dawn (101). In agreement with these observations, we have recently demonstrated that the circadian clock can coordinate the temporal translation of a subset of mRNAs involved in ribosome biogenesis by controlling the transcription of translation initiation factors, as well as the rhythmic activation of signaling pathways involved in their regulation (102). Later experiments using ribosome profiling allowed us to show that two main classes of mRNA are indeed subjected to rhythmic translation: the 5′-terminal oligopyrimidine tract (5′-TOP) mRNAs, translated in a TORC1-dependent manner and involved in ribosome biogenesis (103), and the translation initiator of short 5′ UTR (TISU) motif harboring mRNA coding mostly for mitochondrial proteins (104). Although both circadian clock and feeding rhythms appeared to be involved in the translational regulation of the latter class, only feeding rhythms seem to regulate the translation of 5′-TOP mRNA (83).

However, additional regulations of rRNA synthesis and maturation, as well as ribosome assembly, are subjected to rhythmic regulation potentially involving the circadian clock (91, 102). Because both size and organization of the nucleolus are directly related to ribosome production (105), it is notable that the size of the nucleolus in sympathetic neurons follows a diurnal pattern with a maximum in the middle of the dark period (106), in synchrony with the observed accumulation of ribosomal proteins in the liver.

Another level of circadian translational regulation has been described. While it has already been shown that the size of the poly(A) tail of some mRNA is subject to circadian variation (107), Kojima et al. showed that around 2% of the mRNAs expressed in mouse liver exhibit a rhythmic size of their poly(A) tail, even though their steady-state mRNA levels are not rhythmic (108). The size of the poly(A) tail is under the control of rhythmic cytoplasmic polyadenylation, regulated in part through the rhythmic expression of cytoplasmic polyadenylation element-binding proteins. Interestingly, they show that the rhythm of the length of the poly(A) tail of these mRNAs correlates with the rhythmic expression of the corresponding encoded proteins, with a several-hour delay between the time of longest poly(A) tail and the highest protein levels. Importantly, this study demonstrates that the rhythmic polyadenylation status of mRNAs can result in rhythmic protein expression independent of the steady-state levels of the mRNA. This rhythmic poly(A) length could likely be under the regulation of the clock-controlled NOCTURNIN (NOC) deadenylase (109). Remarkably, a search for NOC-regulated polyadenylated genes revealed that ribosome biogenesis and mitochondrial oxidative phosphorylation are the primary functions regulated by NOC, showing a convergent regulation of these pathways by the circadian clock (110). Therefore, it is not surprising that impaired mitochondrial activity is observed in several circadian clock-mutant mice (111–113).

## Characterization of the Circadian Proteomes and Posttranslational Regulations

During the previous decade and until recently, the literature in large-scale circadian expression studies relied on genomic approach technologies, and proteomics played a limited role due to technological limitations. Pioneer circadian proteomic studies relied on 2-dimensional gel electrophoresis (2D-GE) followed by mass spectrometry (MS). This approach allowed separation of complex protein mixtures and visualization of expression pattern changes in diverse conditions such as different times of the day. This technique was successfully applied to the study of circadian protein expression of organs such as the SCN (114), the rat pineal gland (115), and the mouse retina (116).

The first breakthrough came from the laboratory of M. Hastings where the liver circadian proteome was investigated. Their 2D-GE analyses detected 642 protein spots, of which 60 showed significant rhythmicity. MS identified 39 rhythmic proteins originating from 29 unique genes (86). Using the same experimental design, an exploration of the mouse SCN proteome was conducted in the same laboratory and 34 proteins exhibiting significant rhythmic patterns were identified. This rhythmic proteome was highly enriched with proteins implicated in vesicle trafficking and synaptic vesicle recycling. Moreover, both studies showed a small fraction of corresponding rhythmic mRNA, highlighting the importance of posttranscriptional regulation (86, 117). This work was published just after the study by Hatcher et al. (118) and before the study by Lee et al. (119), both of which characterized the circadian SCN peptides released by MS. Subsequently, an automated and integrated proteomics platform was designed to study the effect of light stimulation on the murine SCN proteome, and, from the 2,131 proteins identified, 387 were shown to be light regulated (120).

Recently, new quantitative proteomic techniques have been developed, from label-free (LF) proteomics to stable-isotope labeling by amino acids in cell culture (SILAC) (121). Accordingly, these tools were used to decipher the rhythmic circadian proteome in both SCN and liver. LF proteomics was used to quantify circadian-related peptides from the SCN (122), and more recently, SILAC was applied to quantify the rhythmic SCN proteome (87). We have used *in vivo* SILAC in mice (123) to characterize the diurnal oscillations of the liver proteome. We identified 5,827 proteins in total protein liver extract, of which 6% were rhythmic and accumulated mostly in the morning and during the night. Half of the rhythmic proteome did not display corresponding rhythmic mRNAs, and the rhythmicity of this group, in which secreted proteins were overrepresented, appeared to be clock independent. This indicates that feeding behavior might determine the rhythm of circulating proteins in the blood (89). This discovery was in accordance with previous data from the study by Martino et al. showing no association between the plasma proteome and the mouse liver transcriptome (124). A parallel study also using an *in vivo* SILAC approach but in constant darkness drew similar conclusions (88). This absence of rhythmicity at the mRNA level for nevertheless cyclic proteins suggests that the regulation of the rhythmic proteome results from posttranscriptional and even posttranslational modification events, since the circadian clock-regulated translation impacts only a limited subset of genes (83, 125, 126).

A caveat when working with total proteomes is their high level of complexity, which can result in difficulties detecting important proteins expressed at a low level such as the core clock proteins, transcription factors, or organelle-specific proteins. To bypass proteome complexity and enhance its resolution, initial organelle biochemical fractionations can be performed before applying quantitative proteomics. This strategy was applied to quantify the mitochondrial proteome using LF quantitative proteomics. Thirty-eight percent of the mitochondrial proteins were cycling, the majority peaking during the early light phase, with low corresponding rhythmic mRNA. These data highlighted the role of posttranscriptional regulation orchestrated by the clock and feeding rhythms in the regulation of mitochondrial function such as fatty acid oxidation. The rhythmic mitochondrial proteome was also correlated with the expression of the TIM/TOM complex, suggesting that protein entry in the mitochondria was temporally framed (90). Alternatively, mitochondrial fission fusion and autophagy, orchestrated by the circadian clock, might also influence the dynamics of the mitochondrial proteome (112).

By using *in vivo* SILAC, we recently produced unprecedented nuclear quantitative proteomic data. Indeed, among the 4,035 nuclear proteins quantified, more than 500 were highly rhythmic, including all the core clock components along with the clock-controlled transcription factors. These findings are in accordance with the absolute quantification of circadian clock proteins published in parallel (91, 127). The rhythmic nuclear proteins were mainly controlled at the posttranscriptional level and were members of complexes displaying robust diurnal nuclear accumulation. These complexes were involved in ribosome biogenesis and assembly (102), as well as DNA repair (128) and transcriptional regulation. In fact, we quantified the rhythmic temporal accumulation of around 100 transcription factors and transcriptional coregulators and provided new insights into the diurnal regulatory landscape in liver nuclei (91).

Reddy et al. already predicted that posttranslational modifications play a role in the regulation of the rhythmic proteome. They identified two different phosphorylated forms of peroxiredoxin 6 displaying antiphasic levels of phosphorylation and the other one being in phase with the transcript (86). This observation led to the discovery that peroxiredoxins undergo circadian redox cycles in association with oscillations in NADH and NADPH, independent of transcription (129, 130). This posttranslational clock is also conserved in all domains of life, probably as a sensor of rhythmic metabolism (131). NADPH, in eukaryotic cells, is provided by the pentose phosphate pathway, which was recently shown to regulate circadian redox oscillations and influence transcriptional oscillations (132). In response to daily changes in nutrient availability and physiological states, numerous posttranslational modifications of the circadian clock have been identified, and modifications such as phosphorylation, ubiquitination, acetylation, O-GlcNacylation, and SUMOylation were shown to have a direct role in fine-tuning the timing of the molecular circadian clock and related metabolic pathways [for reviews, see Ref. (2, 133)].

Phosphorylation has already been described as the base of the circadian clock system in cyanobacteria (134) and is by far the mostly studied posttranslational modification. Moreover, among signaling pathways rhythmically activated by phosphorylation in mouse liver, AMPK and ERK pathways are activated during the day, corresponding to the fasting period (102, 135), whereas AKT and TORC1 pathways are activated during the night, corresponding to the feeding period (102). These results were recently corroborated by total phosphoproteomics analysis, emphasizing the impact of the rhythmic activation of these pathways on the general regulation of metabolism and physiology (90). In parallel, nuclear phosphoproteomic analysis also underlined the alignment of the cell cycle and the circadian clock and its potential role in the regulation of hepatocyte ploidy (91). Both studies also identified rhythmic phosphorylation sites within the core clock proteins, such as the serine 446 and serine 440/441 of CLOCK implicated in regulating its transcriptional activity (136). We found that the serine 42 of BMAL1 is rhythmically phosphorylated in the nucleus (91). This phosphorylation event, under the control of the insulin-AKT-mTOR pathway, reduces the nuclear accumulation of BMAL1 and stabilizes the protein in the cytosol (136, 137). By finely tuning the clock, this mechanism could be one potential contributor to the beneficial effects of restricted feeding. Although the relative impact of feeding entrainable oscillators and circadian rhythms on these rhythmic pathway activations is still poorly described and understood, evidence suggested strong interactions between the circadian clock and metabolism might be key for rhythmic activation of the TORC1 pathway (138, 139).

Beyond phosphorylation, acetylation oscillations have also been shown to play an important role in the rhythmic regulation of liver physiology. In the nucleus, the SIRT1 deacetylase plays a critical role in the organization of the circadian clock in both SCN and peripheral tissues (140–142). In parallel, both SIRT6 and HDAC3 are more involved in the transcriptional regulation of rhythmic metabolism, in particular lipid metabolism (143, 144). SIRT7, another nuclear located deacetylase, was also linked *in vivo* to lipid metabolism, since its expression alleviates ER stress and prevents fatty liver development (145). Moreover, in mouse liver, it specifically locates the promoter of ribosomal proteins for transcriptional silencing and its activity is potentiated by ribosomal RNAs (145, 146). We showed that SIRT7 accumulation is rhythmic in the nucleus, in phase with rRNAs and in opposite phase with translation (91, 102). Hence, in mammals, SIRT7 may be certainly an important contributor to the rhythmic regulation of ribosomal biogenesis and result in rhythmic protein translation. In the cytoplasm, SIRT2 regulates the pentose phosphate pathway by deacetylating glucose-6-phosphate dehydrogenase, which might be important for the regulation of circadian redox oscillations (132, 147). In accordance with our nuclear proteomic data, SIRT2 transiently peaks in the nucleus during mitosis where it has several functions such as the regulation of nuclear envelope reassembly (91, 148).

To date, one large-scale proteomic study looked at the circadian acetylome, finding 306 acetylation sites within 179 proteins (highly enriched in mitochondrial proteins), of which a few sites showed disrupted rhythm in CLOCK-mutant animals (149). The circadian activity of Sirtuins is mainly the consequence of the circadian clock-dependent synthesis of its cofactor NAD^+^ through the NAD^+^ salvage pathway (150, 151). Therefore, this rhythmic NAD^+^ synthesis controls SIRT3 activity in the mitochondria and is involved in the rhythmic mitochondrial activity controlled by the circadian clock (111). Our recent characterization of the rhythmic acetylome identified around 100 rhythmic acetylation sites in mouse liver, mostly originating from mitochondrial proteins. These rhythmic mitochondrial acetylations are correlated with a SIRT3-dependant deacetylation process (unpublished observation).

## The Interaction Between Host Circadian Rhythms and Gut Microbiota

In addition to the regulation of transcription, translation and posttranslational modifications by the circadian clock, recent studies also point out the impact of circadian and feeding rhythms on host gut microbiota adding a new layer of complexity to this interplay. The gut microbiome is a complex assembly of more than a thousand microorganisms, mostly commensal bacteria. Gut microbiota play an important role in gut physiology and host metabolism (152). A decrease in microbiota diversity has been associated with metabolic diseases that include obesity and type 2 diabetes (153). Recent studies show that both the composition and the activity of the gut microbiome (154–157), as well as its adherence to the intestinal epithelium (158), are highly dynamic and exhibit a diurnal pattern. This rhythm appears to be dependent on a functional circadian clock in the host (154, 156). Indeed, the cyclic changes in composition and diversity of gut microbiota are absent in mouse models in which clock function is genetically compromised. However, these absent rhythms can be restored by a time-restricted feeding regimen, strongly suggesting that the main driver of daily fluctuation of gut microbiota composition is feeding rhythm (154, 155, 158, 159). Conversely, gut microbiota has been reported to feedback on host clock gene expression. Germ-free or antibiotic-treated mice that are devoid of gut microbiota were shown to have perturbation of the liver and the intestinal circadian clock. However, the reported effects on peripheral clock gene expression are highly variable, ranging from disruption of clock gene expression in the ileum and colon (160), to rather mild changes in phases and amplitude (157, 161) and virtually no alteration in mouse liver (158). Future studies will evaluate the impact of gut microbiota on host circadian rhythms in more detail to improve our understanding of the mechanism underlying this interaction. Moreover, gut microbiota has also been described as an important modulator of brain function and behavior, including feeding behavior and appetite control (162). It will be interesting to understand how this layer of bacteria–host communication feeds into interaction between the host’s circadian clock and gut microbiota.

## Conclusion: Impact of Feeding Rhythms on Metabolic Health

**Figure 2:**
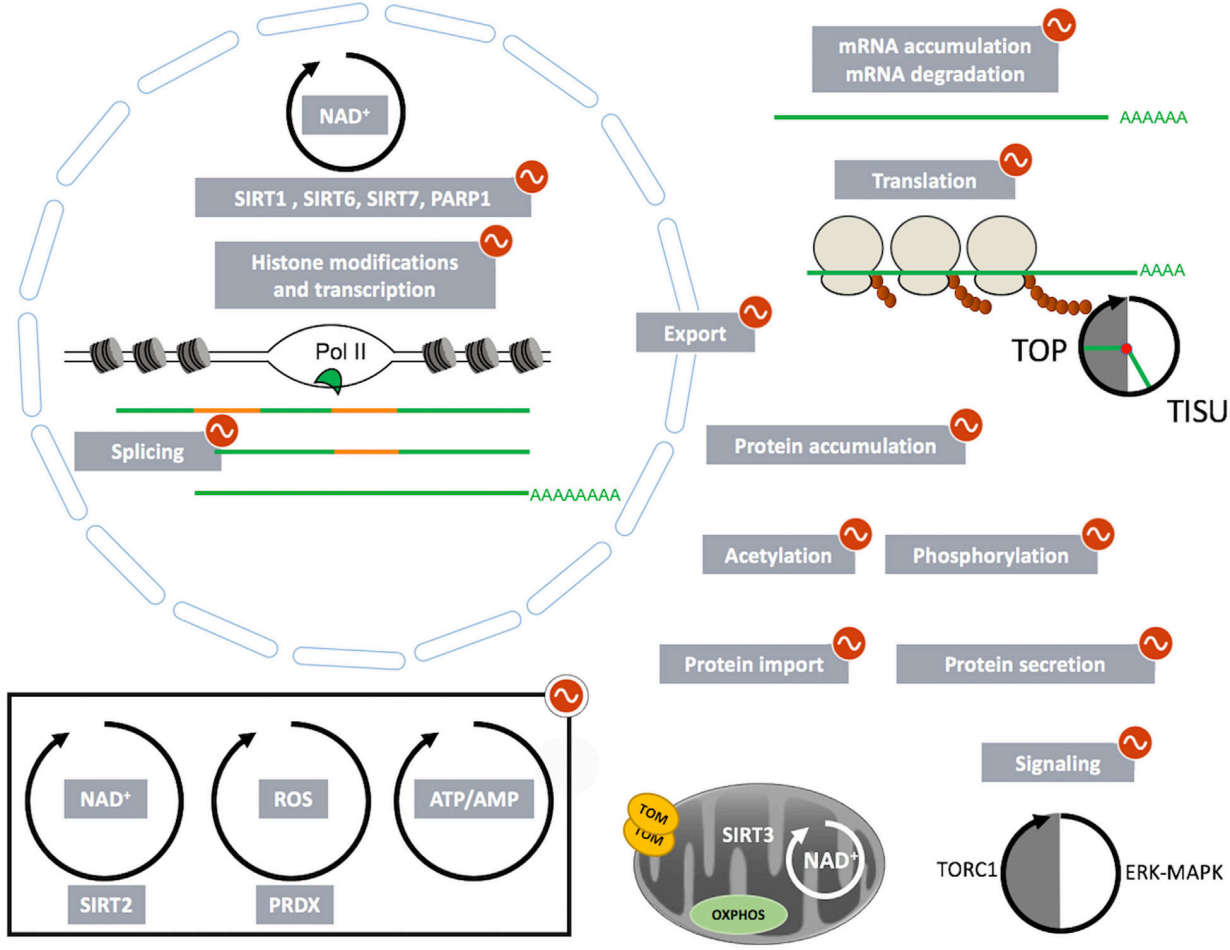
**The multisteps regulated by circadian and feeding rhythms involved in genes product expression: from mRNA transcription to posttranslational modifications and cell trafficking**.

In this review, we summarized the impact of circadian and feeding rhythms not only on rhythmic transcriptional regulations but also on rhythmic posttranscriptional events orchestrated by these tightly interconnected rhythms (Figure 2). Interestingly, imposed feeding rhythms have the capacity to synchronize or increase oscillations in models characterized by decreased amplitude of metabolic and feeding rhythms. For example, imposed day feeding is able to restore liver rhythmic gene expression in clock-deficient mice (39). In addition, deleterious metabolic effect of high-fat diet has been successfully counteract by imposed restricted feeding during the night, the active phase of the animals (85, 163–166). Indeed, limiting access to a high-fat diet during the night increased the amplitude of metabolic rhythms and reduced the health consequences of a high-fat diet without changing the global quantity of ingested calories. This regimen also limits the deleterious impact of high-fat diet-induced obesity on gut microbiota (155, 159). Moreover, the same kind of observation is also true for humans (167) as imposed feeding patterns improved weight loss under calorie restriction (168). Therefore, these studies constitute the basis of chrononutrition, an approach that aims to improve metabolic health through the synchronization of the circadian clock with downstream feeding and sleeping cycles strongly impacted by living environment (169).

## Author Contributions

All authors listed have made substantial, direct, and intellectual contribution to the work and approved it for publication.

## Conflict of Interest Statement

Authors are employees of Nestlé Institute of Health Sciences SA.

## References

[1] Panda S Circadian physiology of metabolism. Science (2016) 354:1008 10.1126/science.aah4967 27885007 PMC7261592

[2] Hirano A Fu YH Ptacek LJ. The intricate dance of post-translational modifications in the rhythm of life. Nat Struct Mol Biol (2016) 23:1053–60. 10.1038/nsmb.3326 27922612

[3] Papazyan R Zhang Y Lazar MA. Genetic and epigenomic mechanisms of mammalian circadian transcription. Nat Struct Mol Biol (2016) 23:1045–52. 10.1038/nsmb.3324 27922611 PMC5497498

[4] Stephan FK Zucker I. Circadian rhythms in drinking behavior and locomotor activity of rats are eliminated by hypothalamic lesions. Proc Natl Acad Sci U S A (1972) 69:1583–6. 10.1073/pnas.69.6.1583 4556464 PMC426753

[5] Ralph MR Foster RG Davis FC Menaker M. Transplanted suprachiasmatic nucleus determines circadian period. Science (1990) 247:975–8. 10.1126/science.2305266 2305266

[6] Sujino M Masumoto KH Yamaguchi S van der Horst GTJ Okamura H Inouye ST. Suprachiasmatic nucleus grafts restore circadian behavioral rhythms of genetically arrhythmic mice. Curr Biol (2003) 13:664–8. 10.1016/S0960-9822(03)00222-7 12699623

[7] Berson DM Dunn FA Takao M. Phototransduction by retinal ganglion cells that set the circadian clock. Science (2002) 295:1070–3. 10.1126/science.1067262 11834835

[8] Hattar S Liao HW Takao M Berson DM Yau KW. Melanopsin-containing retinal ganglion cells: architecture, projections, and intrinsic photosensitivity. Science (2002) 295:1065–70. 10.1126/science.1069609 11834834 PMC2885915

[9] Chen SK Badea TC Hattar S. Photoentrainment and pupillary light reflex are mediated by distinct populations of ipRGCs. Nature (2011) 476:92–5. 10.1038/nature10206 21765429 PMC3150726

[10] Guler AD Ecker JL Lall GS Haq S Altimus CM Liao HW Melanopsin cells are the principal conduits for rod-cone input to non-image-forming vision. Nature (2008) 453:102–5. 10.1038/nature06829 18432195 PMC2871301

[11] Inagaki N Honma S Ono D Tanahashi Y Honma KI. Separate oscillating cell groups in mouse suprachiasmatic nucleus couple photoperiodically to the onset and end of daily activity. Proc Natl Acad Sci U S A (2007) 104:7664–9. 10.1073/pnas.0607713104 17463091 PMC1857228

[12] Liu AC Welsh DK Ko CH Tran HG Zhang EE Priest AA Intercellular coupling confers robustness against mutations in the SCN circadian clock network. Cell (2007) 129:605–16. 10.1016/j.cell.2007.02.047 17482552 PMC3749832

[13] Welsh DK Takahashi JS Kay SA. Suprachiasmatic nucleus: cell autonomy and network properties. Annu Rev Physiol (2010) 72:551–77. 10.1146/annurev-physiol-021909-135919 20148688 PMC3758475

[14] Herzog ED. Neurons and networks in daily rhythms. Nat Rev Neurosci (2007) 8:790–802. 10.1038/nrn2215 17882255

[15] Welsh DK Logothetis DE Meister M Reppert SM. Individual neurons dissociated from rat suprachiasmatic nucleus express independently phased circadian firing rhythms. Neuron (1995) 14:697–706. 10.1016/0896-6273(95)90214-7 7718233

[16] Ginty DD Kornhauser JM Thompson MA Bading H Mayo KE Takahashi JS Regulation of CREB phosphorylation in the suprachiasmatic nucleus by light and a circadian clock. Science (1993) 260:238–41. 10.1126/science.8097062 8097062

[17] Shigeyoshi Y Taguchi K Yamamoto S Takekida S Yan L Tei H Light-induced resetting of a mammalian circadian clock is associated with rapid induction of the mPer1 transcript. Cell (1997) 91:1043–53. 10.1016/S0092-8674(00)80494-8 9428526

[18] Partch CL Green CB Takahashi JS. Molecular architecture of the mammalian circadian clock. Trends Cell Biol (2014) 24:90–9. 10.1016/j.tcb.2013.07.002 23916625 PMC3946763

[19] Stratmann M Suter DM Molina N Naef F Schibler U. Circadian Dbp transcription relies on highly dynamic BMAL1-CLOCK interaction with E boxes and requires the proteasome. Mol Cell (2012) 48:277–87. 10.1016/j.molcel.2012.08.012 22981862

[20] Gerhart-Hines Z Lazar MA. Rev-erbα and the circadian transcriptional regulation of metabolism. Diabetes Obes Metab (2015) 17:12–6. 10.1111/dom.12510 26332963 PMC4562061

[21] Preitner N Damiola F Lopez-Molina L Zakany J Duboule D Albrecht U The orphan nuclear receptor REV-ERBα controls circadian transcription within the positive limb of the mammalian circadian oscillator. Cell (2002) 110:251–60. 10.1016/S0092-8674(02)00825-5 12150932

[22] Sato TK Panda S Miraglia LJ Reyes TM Rudic RD McNamara P A functional genomics strategy reveals Rora as a component of the mammalian circadian clock. Neuron (2004) 43:527–37. 10.1016/j.neuron.2004.07.018 15312651

[23] Honma S Kawamoto T Takagi Y Fujimoto K Sato F Noshiro M *Dec1* and *Dec2* are regulators of the mammalian molecular clock. Nature (2002) 419:841–4. 10.1038/nature01123 12397359

[24] Kato Y Kawamoto T Fujimoto K Noshiro M. DEC1/STRA13/SHARP2 and DEC2/SHARP1 coordinate physiological processes, including circadian rhythms in response to environmental stimuli. Curr Top Dev Biol (2014) 110:339–72. 10.1016/B978-0-12-405943-6.00010-5 25248482

[25] Gachon F. Physiological function of PARbZip circadian clock-controlled transcription factors. Ann Med (2007) 39:562–71. 10.1080/07853890701491034 17852034

[26] Inouye ST Kawamura H Persistence of circadian rhythmicity in a mammalian hypothalamic “island” containing the suprachiasmatic nucleus. Proc Natl Acad Sci U S A (1979) 76:5962–6. 10.1073/pnas.76.11.5962 293695 PMC411773

[27] Yamazaki S Numano R Abe M Hida A Takahashi R Ueda M Resetting central and peripheral circadian oscillators in transgenic rats. Science (2000) 288:682–5. 10.1126/science.288.5466.682 10784453

[28] Yoo SH Yamazaki S Lowrey PL Shimomura K Ko CH Buhr ED PERIOD2::LUCIFERASE real-time reporting of circadian dynamics reveals persistent circadian oscillations in mouse peripheral tissues. Proc Natl Acad Sci U S A (2004) 101:5339–46. 10.1073/pnas.0308709101 14963227 PMC397382

[29] Marcheva B Ramsey KM Buhr ED Kobayashi Y Su H Ko CH Disruption of the clock components CLOCK and BMAL1 leads to hypoinsulinaemia and diabetes. Nature (2010) 466:627–31. 10.1038/nature09253 20562852 PMC2920067

[30] Paschos GK Ibrahim S Song WL Kunieda T Grant G Reyes TM Obesity in mice with adipocyte-specific deletion of clock component *Arntl*. Nat Med (2012) 18:1768–77. 10.1038/nm.2979 23142819 PMC3782286

[31] Lamia KA Storch KF Weitz CJ. Physiological significance of a peripheral tissue circadian clock. Proc Natl Acad Sci U S A (2008) 105:15172–7. 10.1073/pnas.0806717105 18779586 PMC2532700

[32] Kornmann B Schaad O Bujard H Takahashi JS Schibler U. System-driven and oscillator-dependent circadian transcription in mice with a conditionally active liver clock. PLoS Biol (2007) 5:e34. 10.1371/journal.pbio.0050034 17298173 PMC1783671

[33] Balsalobre A Brown SA Marcacci L Tronche F Kellendonk C Reichardt HM Resetting of circadian time in peripheral tissues by glucocorticoid signaling. Science (2000) 289:2344–7. 10.1126/science.289.5488.2344 11009419

[34] Brown SA Zumbrunn G Fleury-Olela F Preitner N Schibler U. Rhythms of mammalian body temperature can sustain peripheral circadian clocks. Curr Biol (2002) 12:1574–83. 10.1016/S0960-9822(02)01145-4 12372249

[35] Vujović N Davidson AJ Menaker M Sympathetic input modulates, but does not determine, phase of peripheral circadian oscillators. Am J Physiol Regul Integr Comp Physiol (2008) 295:R355–60. 10.1152/ajpregu.00498.2007 18434440 PMC2494822

[36] Damiola F Le Minh N Preitner N Kornmann B Fleury-Olela F Schibler U. Restricted feeding uncouples circadian oscillators in peripheral tissues from the central pacemaker in the suprachiasmatic nucleus. Genes Dev (2000) 14:2950–61. 10.1101/gad.183500 11114885 PMC317100

[37] Nagai K Nishio T Nakagawa H Nakamura S Fukuda Y Effect of bilateral lesions of the suprachiasmatic nuclei on the circadian rhythm of food-intake. Brain Res (1978) 142:384–9. 10.1016/0006-8993(78)90648-0 630395

[38] Turek FW Joshu C Kohsaka A Lin E Ivanova G McDearmon E Obesity and metabolic syndrome in circadian clock mutant mice. Science (2005) 308:1043–5. 10.1126/science.1108750 15845877 PMC3764501

[39] Vollmers C Gill S DiTacchio L Pulivarthy SR Le HD Panda S Time of feeding and the intrinsic circadian clock drive rhythms in hepatic gene expression. Proc Natl Acad Sci U S A (2009) 106:21453–21458. 10.1073/pnas.0909591106 19940241 PMC2795502

[40] Stokkan KA Yamazaki S Tei H Sakaki Y Menaker M. Entrainment of the circadian clock in the liver by feeding. Science (2001) 291:490–3. 10.1126/science.291.5503.490 11161204

[41] Hara R Wan K Wakamatsu H Aida R Moriya T Akiyama M Restricted feeding entrains liver clock without participation of the suprachiasmatic nucleus. Genes Cells (2001) 6:269–78. 10.1046/j.1365-2443.2001.00419.x 11260270

[42] Saini C Liani A Curie T Gos P Kreppel F Emmenegger Y Real-time recording of circadian liver gene expression in freely moving mice reveals the phase-setting behavior of hepatocyte clocks. Genes Dev (2013) 27:1526–36. 10.1101/gad.221374.113 23824542 PMC3713432

[43] Le Minh N Damiola F Tronche F Schutz G Schibler U. Glucocorticoid hormones inhibit food-induced phase-shifting of peripheral circadian oscillators. EMBO J (2001) 20:7128–36. 10.1093/emboj/20.24.7128 11742989 PMC125339

[44] Buijs RM Wortel J Van Heerikhuize JJ Feenstra MGP Ter Horst GJ Romijn HJ Anatomical and functional demonstration of a multisynaptic suprachiasmatic nucleus adrenal (cortex) pathway. Eur J Neurosci (1999) 11:1535–44. 10.1046/j.1460-9568.1999.00575.x 10215906

[45] Ishida A Mutoh T Ueyama T Bando H Masubuchi S Nakahara D Light activates the adrenal gland: timing of gene expression and glucocorticoid release. Cell Metab (2005) 2:297–307. 10.1016/j.cmet.2005.09.009 16271530

[46] Reddy AB Maywood ES Karp NA King VM Inoue Y Gonzalez FJ Glucocorticoid signaling synchronizes the liver circadian transcriptome. Hepatology (2007) 45:1478–88. 10.1002/hep.21571 17538967

[47] Yamamoto T Nakahata Y Tanaka M Yoshida M Soma H Shinohara K Acute physical stress elevates mouse *period1* mRNA expression in mouse peripheral tissues via a glucocorticoid-responsive element. J Biol Chem (2005) 280:42036–42043. 10.1074/jbc.M509600200 16249183

[48] So AYL Bernal TU Pillsbury ML Yamamoto KR Feldman BJ. Glucocorticoid regulation of the circadian clock modulates glucose homeostasis. Proc Natl Acad Sci U S A (2009) 106:17582–7. 10.1073/pnas.0909733106 19805059 PMC2757402

[49] Weger BD Weger M Görling B Schink A Gobet C Keime C Extensive regulation of diurnal transcription and metabolism by glucocorticoids. PLoS Genet (2016) 12:e1006512. 10.1371/journal.pgen.1006512 27941970 PMC5191836

[50] Buhr ED Yoo SH Takahashi JS. Temperature as a universal resetting cue for mammalian circadian oscillators. Science (2010) 330:379–85. 10.1126/science.1195262 20947768 PMC3625727

[51] Saini C Morf J Stratmann M Gos P Schibler U. Simulated body temperature rhythms reveal the phase-shifting behavior and plasticity of mammalian circadian oscillators. Genes Dev (2012) 26:567–80. 10.1101/gad.183251.111 22379191 PMC3315118

[52] Dibner C Sage D Unser M Bauer C d’Eysmond T Naef F Circadian gene expression is resilient to large fluctuations in overall transcription rates. EMBO J (2009) 28:123–34. 10.1038/emboj.2008.262 19078963 PMC2634731

[53] Reinke H Saini C Fleury Olela F Dibner C Benjamin IJ Schibler U. Differential display of DNA-binding proteins reveals heat-shock factor 1 as a circadian transcription factor. Genes Dev (2008) 22:331–45. 10.1101/gad.453808 18245447 PMC2216693

[54] Krieger DT Hauser H Krey LC. Suprachiasmatic nuclear lesions do not abolish food-shifted circadian adrenal and temperature rhythmicity. Science (1977) 197:398. 10.1126/science.877566 877566

[55] Stephan FK Swann JM Sisk CL Entrainment of circadian rhythms by feeding schedules in rats with suprachiasmatic lesions. Behav Neural Biol (1979) 25:545–54. 10.1016/S0163-1047(79)90415-1 464989

[56] Gotic I Omidi S Fleury-Olela F Molina N Naef F Schibler U. Temperature regulates splicing efficiency of the cold-inducible RNA-binding protein gene *Cirbp*. Genes Dev (2016) 30:2005–17. 10.1101/gad.287094.116 27633015 PMC5066242

[57] Krieger DT Food and water restriction shifts corticosterone, temperature, activity and brain amine periodicity. Endocrinology (1974) 95:1195–201. 10.1210/endo-95-5-1195 4426285

[58] Stephan FK The “other” circadian system: food as a Zeitgeber. J Biol Rhythms (2002) 17:284–92. 10.1177/074873002129002591 12164245

[59] Pitts S Perone E Silver R. Food-entrained circadian rhythms are sustained in arrhythmic *Clk*/*Clk* mutant mice. Am J Physiol Regul Integr Comp Physiol (2003) 285:R57–67. 10.1152/ajpregu.00023.2003 12649127 PMC3837688

[60] Storch KF Weitz CJ. Daily rhythms of food-anticipatory behavioral activity do not require the known circadian clock. Proc Natl Acad Sci U S A (2009) 106:6808–13. 10.1073/pnas.0902063106 19366674 PMC2666092

[61] Laermans J Vancleef L Tack J Depoortere I. Role of the clock gene Bmal1 and the gastric ghrelin-secreting cell in the circadian regulation of the ghrelin-GOAT system. Sci Rep (2015) 5:16748. 10.1038/srep16748 26576661 PMC4649743

[62] LeSauter J Hoque N Weintraub M Pfaff DW Silver R. Stomach ghrelin-secreting cells as food-entrainable circadian clocks. Proc Natl Acad Sci U S A (2009) 106:13582–7. 10.1073/pnas.0906426106 19633195 PMC2726387

[63] Landgraf D Tsang AH Leliavski A Koch CE Barclay JL Drucker DJ Oxyntomodulin regulates resetting of the liver circadian clock by food. Elife (2015) 4:e06253. 10.7554/eLife.06253 25821984 PMC4426666

[64] Chavan R Feillet C Costa SSF Delorme JE Okabe T Ripperger JA Liver-derived ketone bodies are necessary for food anticipation. Nat Commun (2016) 7:10580. 10.1038/ncomms10580 26838474 PMC4742855

[65] Feillet CA Ripperger JA Magnone MC Dulloo A Albrecht U Challet E. Lack of food anticipation in *Per2* mutant mice. Curr Biol (2006) 16:2016–22. 10.1016/j.cub.2006.08.053 17055980

[66] Kettner NM Mayo SA Hua J Lee C Moore DD Fu L. Circadian dysfunction induces leptin resistance in mice. Cell Metab (2015) 22:448–59. 10.1016/j.cmet.2015.06.005 26166747 PMC4558341

[67] Koike N Yoo SH Huang HC Kumar V Lee C Kim TK Transcriptional architecture and chromatin landscape of the core circadian clock in mammals. Science (2012) 338:349–54. 10.1126/science.1226339 22936566 PMC3694775

[68] Ripperger JA Schibler U. Rhythmic CLOCK-BMAL1 binding to multiple E-box motifs drives circadian *Dbp* transcription and chromatin transitions. Nat Genet (2006) 38:369–74. 10.1038/ng1738 16474407

[69] Panda S Antoch MP Miller BH Su AI Schook AB Straume M Coordinated transcription of key pathways in the mouse by the circadian clock. Cell (2002) 109:307–20. 10.1016/S0092-8674(02)00722-5 12015981

[70] Storch KF Lipan O Leykin I Viswanathan N Davis FC Wong WH Extensive and divergent circadian gene expression in liver and heart. Nature (2002) 417:78–83. 10.1038/nature744 11967526

[71] Ueda HR Chen W Adachi A Wakamatsu H Hayashi S Takasugi T A transcription factor response element for gene expression during circadian night. Nature (2002) 418:534–9. 10.1038/nature00906 12152080

[72] Grimaldi B Bellet MM Katada S Astarita G Hirayama J Amin RH PER2 controls lipid metabolism by direct regulation of PPARg. Cell Metab (2010) 12:509–20. 10.1016/j.cmet.2010.10.005 21035761 PMC4103168

[73] Schmutz I Ripperger JA Baeriswyl-Aebischer S Albrecht U. The mammalian clock component PERIOD2 coordinates circadian output by interaction with nuclear receptors. Genes Dev (2010) 24:345–57. 10.1101/gad.564110 20159955 PMC2816734

[74] Cho H Zhao X Hatori M Yu RT Barish GD Lam MT Regulation of circadian behaviour and metabolism by REV-ERB-α and REV-ERB-β. Nature (2012) 485:123–7. 10.1038/nature11048 22460952 PMC3367514

[75] Zhang Y Fang B Emmett MJ Damle M Sun Z Feng D Discrete functions of nuclear receptor Rev-erbα couple metabolism to the clock. Science (2015) 348:1488–92. 10.1126/science.aab3021 26044300 PMC4613749

[76] Lamia KA Papp SJ Yu RT Barish GD Uhlenhaut NH Jonker JW Cryptochromes mediate rhythmic repression of the glucocorticoid receptor. Nature (2011) 480:552–6. 10.1038/nature10700 22170608 PMC3245818

[77] Gachon F Leuenberger N Claudel T Gos P Jouffe C Fleury Olela F Proline- and acidic amino acid-rich basic leucine zipper proteins modulate peroxisome proliferator-activated receptor α (PPARα) activity. Proc Natl Acad Sci U S A (2011) 108:4794–9. 10.1073/pnas.1002862108 21383142 PMC3064322

[78] Gilardi F Migliavacca E Naldi A Baruchet M Canella D Le Martelot G Genome-wide analysis of SREBP1 activity around the clock reveals its combined dependency on nutrient and circadian signals. PLoS Genet (2014) 10:e1004155. 10.1371/journal.pgen.1004155 24603613 PMC3945117

[79] Le Martelot G Claudel T Gatfield D Schaad O Kornmann B Sasso GL REV-ERBα participates in circadian SREBP signaling and bile acid homeostasis. PLoS Biol (2009) 7:e1000181 10.1371/journal.pbio.1000181 19721697 PMC2726950

[80] Brewer M Lange D Baler R Anzulovich A. SREBP-1 as a transcriptional integrator of circadian and nutritional cues in the liver. J Biol Rhythms (2005) 20:195–205. 10.1177/0748730405275952 15851526

[81] Menet JS Rodriguez J Abruzzi KC Rosbash M. Nascent-Seq reveals novel features of mouse circadian transcriptional regulation. Elife (2012) 1:e00011. 10.7554/eLife.00011 23150795 PMC3492862

[82] Le Martelot G Canella D Symul L Migliavacca E Gilardi F Liechti R Genome-wide RNA polymerase II profiles and RNA accumulation reveal kinetics of transcription and associated epigenetic changes during diurnal cycles. PLoS Biol (2012) 10:e1001442. 10.1371/journal.pbio.1001442 23209382 PMC3507959

[83] Atger F Gobet C Marquis J Martin E Wang J Weger B Circadian and feeding rhythms differentially affect rhythmic mRNA transcription and translation in mouse liver. Proc Natl Acad Sci U S A (2015) 112:E6579–88. 10.1073/pnas.1515308112 26554015 PMC4664316

[84] Lück S Thurley K Thaben PF Westermark PO. Rhythmic degradation explains and unifies circadian transcriptome and proteome data. Cell Rep (2014) 9:741–51. 10.1016/j.celrep.2014.09.021 25373909

[85] Hatori M Vollmers C Zarrinpar A DiTacchio L Bushong EA Gill S Time-restricted feeding without reducing caloric intake prevents metabolic diseases in mice fed a high-fat diet. Cell Metab (2012) 15:848–60. 10.1016/j.cmet.2012.04.019 22608008 PMC3491655

[86] Reddy AB Karp NA Maywood ES Sage EA Deery M O’Neill JS Circadian orchestration of the hepatic proteome. Curr Biol (2006) 16:1107–15. 10.1016/j.cub.2006.04.026 16753565

[87] Chiang CK Mehta N Patel A Zhang P Ning Z Mayne J The proteomic landscape of the suprachiasmatic nucleus clock reveals large-scale coordination of key biological processes. PLoS Genet (2014) 10:e1004695. 10.1371/journal.pgen.1004695 25330117 PMC4199512

[88] Robles MS Cox J Mann M. In-vivo quantitative proteomics reveals a key contribution of post-transcriptional mechanisms to the circadian regulation of liver metabolism. PLoS Genet (2014) 10:e1004047. 10.1371/journal.pgen.1004047 24391516 PMC3879213

[89] Mauvoisin D Wang J Jouffe C Martin E Atger F Waridel P Circadian clock-dependent and -independent rhythmic proteomes implement distinct diurnal functions in mouse liver. Proc Natl Acad Sci U S A (2014) 111:167–72. 10.1073/pnas.1314066111 24344304 PMC3890886

[90] Neufeld-Cohen A Robles MS Aviram R Manella G Adamovich Y Ladeuix B Circadian control of oscillations in mitochondrial rate-limiting enzymes and nutrient utilization by PERIOD proteins. Proc Natl Acad Sci U S A (2016) 113:E1673–82. 10.1073/pnas.1519650113 26862173 PMC4812734

[91] Wang J Mauvoisin D Martin E Atger F Galindo AN Dayon L Nuclear proteomics uncovers diurnal regulatory landscapes in mouse liver. Cell Metab (2017) 25:102–17. 10.1016/j.cmet.2016.10.003 27818260 PMC5241201

[92] Sweeney BM Haxo FT. Persistence of a photosynthetic rhythm in enucleated *Acetabularia*. Science (1961) 134:1361–3. 10.1126/science.134.3487.1361 17807341

[93] Schweiger E Wallraff HG Schweiger HG. Endogenous circadian rhythm in cytoplasm of *Acetabularia*: influence of the nucleus. Science (1964) 146:658–9. 10.1126/science.146.3644.658 17794040

[94] Woolum JC. A re-examination of the role of the nucleus in generating the circadian rhythm in *Acetabularia*. J Biol Rhythms (1991) 6:129–36. 10.1177/074873049100600203 1773086

[95] Hartwig R Schweiger M Schweiger R Schweiger HG. Identification of a high molecular weight polypeptide that may be part of the circadian clockwork in *Acetabularia*. Proc Natl Acad Sci U S A (1985) 82:6899–902. 10.1073/pnas.82.20.6899 16593618 PMC390795

[96] Hastings JW. The *Gonyaulax* clock at 50: translational control of circadian expression. Cold Spring Harb Symp Quant Biol (2007) 72:141–4. 10.1101/sqb.2007.72.026 18419271

[97] Johnson CH Roeber JF Hastings JW. Circadian changes in enzyme concentration account for rhythm of enzyme activity in *Gonyaulax*. Science (1984) 223:1428–30. 10.1126/science.223.4643.1428 17746055

[98] Morse D Milos PM Roux E Hastings JW. Circadian regulation of bioluminescence in *Gonyaulax* involves translational control. Proc Natl Acad Sci U S A (1989) 86:172–6. 10.1073/pnas.86.1.172 2911566 PMC286426

[99] Mittag M Lee DH Hastings JW. Circadian expression of the luciferin-binding protein correlates with the binding of a protein to the 3’ untranslated region of its mRNA. Proc Natl Acad Sci U S A (1994) 91:5257–61. 10.1073/pnas.91.12.5257 8202478 PMC43973

[100] Fishman B Wurtman RJ Munro HN. Daily rhythms in hepatic polysome profiles and tyrosine transaminase activity: role of dietary protein. Proc Natl Acad Sci U S A (1969) 64:677–82. 10.1073/pnas.64.2.677 4391022 PMC223397

[101] Uchiyama Y Asari A. A morphometric study of the variations in subcellular structures of rat hepatocytes during 24 hours. Cell Tissue Res (1984) 236:305–15. 10.1007/BF00214231 6733756

[102] Jouffe C Cretenet G Symul L Martin E Atger F Naef F The circadian clock coordinates ribosome biogenesis. PLoS Biol (2013) 11:e1001455. 10.1371/journal.pbio.1001455 23300384 PMC3536797

[103] Meyuhas O Kahan T. The race to decipher the top secrets of TOP mRNAs. Biochim Biophys Acta (2015) 1849:801–11. 10.1016/j.bbagrm.2014.08.015 25234618

[104] Sinvani H Haimov O Svitkin Y Sonenberg N Tamarkin-Ben-Harush A Viollet B Translational tolerance of mitochondrial genes to metabolic energy stress involves TISU and eIF1-eIF4GI cooperation in start codon selection. Cell Metab (2015) 21:479–92. 10.1016/j.cmet.2015.02.010 25738462

[105] Hernandez-Verdun D. The nucleolus: a model for the organization of nuclear functions. Histochem Cell Biol (2006) 126:135–48. 10.1007/s00418-006-0212-3 16835752

[106] Seïte R Pébusque MJ. Chronobiological studies on the nucleolus. Chronobiol Int (1985) 2:69–91. 10.3109/07420528509055546 3916701

[107] Robinson BG Frim DM Schwartz WJ Majzoub JA. Vasopressin mRNA in the suprachiasmatic nuclei: daily regulation of polyadenylate tail length. Science (1988) 241:342–4. 10.1126/science.3388044 3388044

[108] Kojima S Sher-Chen EL Green CB. Circadian control of mRNA polyadenylation dynamics regulates rhythmic protein expression. Genes Dev (2012) 26:2724–36. 10.1101/gad.208306.112 23249735 PMC3533077

[109] Stubblefield JJ Terrien J Green CB. Nocturnin: at the crossroads of clocks and metabolism. Trends Endocrinol Metab (2012) 23:326–33. 10.1016/j.tem.2012.03.007 22608110 PMC3389576

[110] Kojima S Gendreau KL Sher-Chen EL Gao P Green CB. Changes in poly(A) tail length dynamics from the loss of the circadian deadenylase Nocturnin. Sci Rep (2015) 5:17059. 10.1038/srep17059 26586468 PMC4653638

[111] Peek CB Affinati AH Ramsey KM Kuo HY Yu W Sena LA Circadian clock NAD^+^ cycle drives mitochondrial oxidative metabolism in mice. Science (2013) 342:1243417. 10.1126/science.1243417 24051248 PMC3963134

[112] Jacobi D Liu S Burkewitz K Kory N Knudsen NH Alexander RK Hepatic Bmal1 regulates rhythmic mitochondrial dynamics and promotes metabolic fitness. Cell Metab (2015) 22:709–20. 10.1016/j.cmet.2015.08.006 26365180 PMC4598294

[113] Woldt E Sebti Y Solt LA Duhem C Lancel S Eeckhoute J Rev-erb-α modulates skeletal muscle oxidative capacity by regulating mitochondrial biogenesis and autophagy. Nat Med (2013) 19:1039–46. 10.1038/nm.3213 23852339 PMC3737409

[114] Fahrenkrug J Hannibal J Honoré B Vorum H. Altered calmodulin response to light in the suprachiasmatic nucleus of PAC1 receptor knockout mice revealed by proteomic analysis. J Mol Neurosci (2005) 25:251–8. 10.1385/JMN:25:3:251 15800378

[115] Møller M Sparre T Bache N Roepstorff P Vorum H Proteomic analysis of day-night variations in protein levels in the rat pineal gland. Proteomics (2007) 7:2009–18. 10.1002/pmic.200600963 17514675

[116] Tsuji T Hirota T Takemori N Komori N Yoshitane H Fukuda M Circadian proteomics of the mouse retina. Proteomics (2007) 7:3500–8. 10.1002/pmic.200700272 17726681

[117] Deery MJ Maywood ES Chesham JE Sládek M Karp NA Green EW Proteomic analysis reveals the role of synaptic vesicle cycling in sustaining the suprachiasmatic circadian clock. Curr Biol (2009) 19:2031–6. 10.1016/j.cub.2009.10.024 19913422

[118] Hatcher NG Atkins N Annangudi SP Forbes AJ Kelleher NL Gillette MU Mass spectrometry-based discovery of circadian peptides. Proc Natl Acad Sci U S A (2008) 105:12527–32. 10.1073/pnas.0804340105 18719122 PMC2518830

[119] Lee JE Atkins N Hatcher NG Zamdborg L Gillette MU Sweedler JV Endogenous peptide discovery of the rat circadian clock: a focused study of the suprachiasmatic nucleus by ultrahigh performance tandem mass spectrometry. Mol Cell Proteomics (2010) 9:285–97. 10.1074/mcp.M900362-MCP200 19955084 PMC2830840

[120] Tian R Alvarez-Saavedra M Cheng HYM Figeys D. Uncovering the proteome response of the master circadian clock to light using an autoproteome system. Mol Cell Proteomics (2011) 10:M110.007252. 10.1074/mcp.M110.007252 21859948 PMC3226397

[121] Bantscheff M Lemeer S Savitski MM Kuster B. Quantitative mass spectrometry in proteomics: critical review update from 2007 to the present. Anal Bioanal Chem (2012) 404:939–65. 10.1007/s00216-012-6203-4 22772140

[122] Lee JE Zamdborg L Southey BR Atkins N Mitchell JW Li M Quantitative peptidomics for discovery of circadian-related peptides from the rat suprachiasmatic nucleus. J Proteome Res (2013) 12:585–93. 10.1021/pr300605p 23256577 PMC3562399

[123] Krüger M Moser M Ussar S Thievessen I Luber CA Forner F SILAC mouse for quantitative proteomics uncovers kindlin-3 as an essential factor for red blood cell function. Cell (2008) 134:353–64. 10.1016/j.cell.2008.05.033 18662549

[124] Martino TA Tata N Bjarnason GA Straume M Sole MJ. Diurnal protein expression in blood revealed by high throughput mass spectrometry proteomics and implications for translational medicine and body time of day. Am J Physiol Regul Integr Comp Physiol (2007) 293:R1430–7. 10.1152/ajpregu.00183.2007 17553849

[125] Jang C Lahens NF Hogenesch JB Sehgal A. Ribosome profiling reveals an important role for translational control in circadian gene expression. Genome Res (2015) 25:1836–47. 10.1101/gr.191296.115 26338483 PMC4665005

[126] Janich P Arpat AB Castelo-Szekely V Lopes M Gatfield D. Ribosome profiling reveals the rhythmic liver translatome and circadian clock regulation by upstream open reading frames. Genome Res (2015) 25:1848–59. 10.1101/gr.195404.115 26486724 PMC4665006

[127] Narumi R Shimizu Y Ukai-Tadenuma M Ode KL Kanda GN Shinohara Y Mass spectrometry-based absolute quantification reveals rhythmic variation of mouse circadian clock proteins. Proc Natl Acad Sci U S A (2016) 113:E3461–7. 10.1073/pnas.1603799113 27247408 PMC4914154

[128] Sancar A Lindsey-Boltz LA Kang TH Reardon JT Lee JH Ozturk N. Circadian clock control of the cellular response to DNA damage. FEBS Lett (2010) 584:2618–25. 10.1016/j.febslet.2010.03.017 20227409 PMC2878924

[129] O’Neill JS Reddy AB. Circadian clocks in human red blood cells. Nature (2011) 469:498–503. 10.1038/nature09702 21270888 PMC3040566

[130] O’Neill JS van Ooijen G Dixon LE Troein C Corellou F Bouget FY Circadian rhythms persist without transcription in a eukaryote. Nature (2011) 469:554–8. 10.1038/nature09654 21270895 PMC3040569

[131] Edgar RS Green EW Zhao Y van Ooijen G Olmedo M Qin X Peroxiredoxins are conserved markers of circadian rhythms. Nature (2012) 485:459–64. 10.1038/nature11088 22622569 PMC3398137

[132] Rey G Valekunja UK Feeney KA Wulund L Milev NB Stangherlin A The pentose phosphate pathway regulates the circadian clock. Cell Metab (2016) 24:462–73. 10.1016/j.cmet.2016.07.024 27546460 PMC5031559

[133] Asher G Sassone-Corsi P. Time for food: the intimate interplay between nutrition, metabolism, and the circadian clock. Cell (2015) 161:84–92. 10.1016/j.cell.2015.03.015 25815987

[134] Johnson CH Mori T Xu Y. A cyanobacterial circadian clockwork. Curr Biol (2008) 18:R816–25. 10.1016/j.cub.2008.07.012 18786387 PMC2585598

[135] Lamia KA Sachdeva UM DiTacchio L Williams EC Alvarez JG Egan DF AMPK regulates the circadian clock by cryptochrome phosphorylation and degradation. Science (2009) 326:437–40. 10.1126/science.1172156 19833968 PMC2819106

[136] Robles MS Humphrey SJ Mann M Phosphorylation is a central mechanism for circadian control of metabolism and physiology. Cell Metab (2017) 25:118–27. 10.1016/j.cmet.2016.10.004 27818261

[137] Dang F Sun X Ma X Wu R Zhang D Chen Y Insulin post-transcriptionally modulates Bmal1 protein to affect the hepatic circadian clock. Nat Commun (2016) 7:12696. 10.1038/ncomms12696 27576939 PMC5013695

[138] Khapre RV Kondratova AA Patel S Dubrovsky Y Wrobel M Antoch MP BMAL1-dependent regulation of the mTOR signaling pathway delays aging. Aging (Albany NY) (2014) 6:48–57. 10.18632/aging.100633 24481314 PMC3927809

[139] Khapre RV Patel SA Kondratova AA Chaudhary A Velingkaar N Antoch MP Metabolic clock generates nutrient anticipation rhythms in mTOR signaling. Aging (Albany NY) (2014) 6:675–89. 10.18632/aging.100686 25239872 PMC4169861

[140] Asher G Gatfield D Stratmann M Reinke H Dibner C Kreppel F SIRT1 regulates circadian clock gene expression through PER2 deacetylation. Cell (2008) 134:317–28. 10.1016/j.cell.2008.06.050 18662546

[141] Nakahata Y Kaluzova M Grimaldi B Sahar S Hirayama J Chen D The NAD^+^-dependent deacetylase SIRT1 modulates CLOCK-mediated chromatin remodeling and circadian control. Cell (2008) 134:329–40. 10.1016/j.cell.2008.07.002 18662547 PMC3526943

[142] Chang HC Guarente L. SIRT1 mediates central circadian control in the SCN by a mechanism that decays with aging. Cell (2013) 153:1448–60. 10.1016/j.cell.2013.05.027 23791176 PMC3748806

[143] Feng D Liu T Sun Z Bugge A Mullican SE Alenghat T Orchestrated by histone deacetylase 3 controls hepatic lipid metabolism. Science (2011) 331:1315–9. 10.1126/science.1198125 21393543 PMC3389392

[144] Masri S Rigor P Cervantes M Ceglia N Sebastian C Xiao C Partitioning circadian transcription by SIRT6 leads to segregated control of cellular metabolism. Cell (2014) 158:659–72. 10.1016/j.cell.2014.06.050 25083875 PMC5472354

[145] Shin J He M Liu Y Paredes S Villanova L Brown K SIRT7 represses Myc activity to suppress ER stress and prevent fatty liver disease. Cell Rep (2013) 5:654–65. 10.1016/j.celrep.2013.10.007 24210820 PMC3888240

[146] Tong Z Wang M Wang Y Kim DD Grenier JK Cao J SIRT7 is an RNA-activated protein lysine deacylase. ACS Chem Biol (2017) 12:300–10. 10.1021/acschembio.6b00954 27997115 PMC5326686

[147] Wang YP Zhou LS Zhao YZ Wang SW Chen LL Liu LX Regulation of G6PD acetylation by SIRT2 and KAT9 modulates NADPH homeostasis and cell survival during oxidative stress. EMBO J (2014) 33:1304–20. 10.1002/embj.201387224 24769394 PMC4194121

[148] Kaufmann T Kukolj E Brachner A Beltzung E Bruno M Kostrhon S SIRT2 regulates nuclear envelope reassembly through ANKLE2 deacetylation. J Cell Sci (2016) 129:4607–21. 10.1242/jcs.192633 27875273 PMC5201015

[149] Masri S Patel VR Eckel-Mahan KL Peleg S Forne I Ladurner AG Circadian acetylome reveals regulation of mitochondrial metabolic pathways. Proc Natl Acad Sci U S A (2013) 110:3339–44. 10.1073/pnas.1217632110 23341599 PMC3587221

[150] Nakahata Y Sahar S Astarita G Kaluzova M Sassone-Corsi P. Circadian control of the NAD^+^ salvage pathway by CLOCK-SIRT1. Science (2009) 324:654–7. 10.1126/science.1170803 19286518 PMC6501775

[151] Ramsey KM Yoshino J Brace CS Abrassart D Kobayashi Y Marcheva B Circadian clock feedback cycle through NAMPT-mediated NAD^+^ biosynthesis. Science (2009) 324:651–4. 10.1126/science.1171641 19299583 PMC2738420

[152] Tremaroli V Backhed F. Functional interactions between the gut microbiota and host metabolism. Nature (2012) 489:242–9. 10.1038/nature11552 22972297

[153] Nieuwdorp M Gilijamse PW Pai N Kaplan LM. Role of the microbiome in energy regulation and metabolism. Gastroenterology (2014) 146:1525–33. 10.1053/j.gastro.2014.02.008 24560870

[154] Thaiss CA Zeevi D Levy M Zilberman-Schapira G Suez J Tengeler AC Transkingdom control of microbiota diurnal oscillations promotes metabolic homeostasis. Cell (2014) 159:514–29. 10.1016/j.cell.2014.09.048 25417104

[155] Zarrinpar A Chaix A Yooseph S Panda S. Diet and feeding pattern affect the diurnal dynamics of the gut microbiome. Cell Metab (2014) 20:1006–17. 10.1016/j.cmet.2014.11.008 25470548 PMC4255146

[156] Liang X Bushman FD FitzGerald GA. Rhythmicity of the intestinal microbiota is regulated by gender and the host circadian clock. Proc Natl Acad Sci U S A (2015) 112:10479–84. 10.1073/pnas.1501305112 26240359 PMC4547234

[157] Leone V Gibbons SM Martinez K Hutchison AL Huang EY Cham CM Effects of diurnal variation of gut microbes and high-fat feeding on host circadian clock function and metabolism. Cell Host Microbe (2015) 17:681–9. 10.1016/j.chom.2015.03.006 25891358 PMC4433408

[158] Thaiss CA Levy M Korem T Dohnalová L Shapiro H Jaitin DA Microbiota diurnal rhythmicity programs host transcriptome oscillations. Cell (2016) 167:1495.e–510.e. 10.1016/j.cell.2016.11.003 27912059

[159] Voigt RM Forsyth CB Green SJ Mutlu E Engen P Vitaterna MH Circadian disorganization alters intestinal microbiota. PLoS One (2014) 9:e97500. 10.1371/journal.pone.0097500 24848969 PMC4029760

[160] Mukherji A Kobiita A Ye T Chambon P. Homeostasis in intestinal epithelium is orchestrated by the circadian clock and microbiota cues transduced by TLRs. Cell (2013) 153:812–27. 10.1016/j.cell.2013.04.020 23663780

[161] Montagner A Korecka A Polizzi A Lippi Y Blum Y Canlet C Hepatic circadian clock oscillators and nuclear receptors integrate microbiome-derived signals. Sci Rep (2016) 6:20127. 10.1038/srep20127 26879573 PMC4754633

[162] Fetissov SO. Role of the gut microbiota in host appetite control: bacterial growth to animal feeding behaviour. Nat Rev Endocrinol (2017) 13:11–25. 10.1038/nrendo.2016.150 27616451

[163] Chaix A Zarrinpar A Miu P Panda S. Time-restricted feeding is a preventative and therapeutic intervention against diverse nutritional challenges. Cell Metab (2014) 20:991–1005. 10.1016/j.cmet.2014.11.001 25470547 PMC4255155

[164] Sundaram S Yan L. Time-restricted feeding reduces adiposity in mice fed a high-fat diet. Nutr Res (2016) 36:603–11. 10.1016/j.nutres.2016.02.005 27188906

[165] Duncan MJ Smith JT Narbaiza J Mueez F Bustle LB Qureshi S Restricting feeding to the active phase in middle-aged mice attenuates adverse metabolic effects of a high-fat diet. Physiol Behav (2016) 167:1–9. 10.1016/j.physbeh.2016.08.027 27586251

[166] Sherman H Genzer Y Cohen R Chapnik N Madar Z Froy O. Timed high-fat diet resets circadian metabolism and prevents obesity. FASEB J (2012) 26:3493–502. 10.1096/fj.12-208868 22593546

[167] Gill S Panda S. A smartphone app reveals erratic diurnal eating patterns in humans that can be modulated for health benefits. Cell Metab (2015) 22:789–98. 10.1016/j.cmet.2015.09.005 26411343 PMC4635036

[168] Jakubowicz D Barnea M Wainstein J Froy O. High caloric intake at breakfast vs. dinner differentially influences weight loss of overweight and obese women. Obesity (Silver Spring) (2013) 21:2504–12. 10.1002/oby.20460 23512957

[169] Oike H Oishi K Kobori M Nutrients, clock genes, and chrononutrition. Curr Nutr Rep (2014) 3:204–12. 10.1007/s13668-014-0082-6 25101217 PMC4118017

